# eMental Healthcare Technologies for Anxiety and Depression in Childhood and Adolescence: Systematic Review of Studies Reporting Implementation Outcomes

**DOI:** 10.2196/mental.9655

**Published:** 2018-06-26

**Authors:** Lori Wozney, Patrick J McGrath, Nicole D Gehring, Kathryn Bennett, Anna Huguet, Lisa Hartling, Michele P Dyson, Amir Soleimani, Amanda S Newton

**Affiliations:** ^1^ Izaak Walton Killam Centre Centre for Research in Family Health Halifax, NS Canada; ^2^ Department of Psychiatry Dalhousie University Halifax, NS Canada; ^3^ Department of Pediatrics Faculty of Medicine & Dentistry University of Alberta Edmonton, AB Canada; ^4^ Offord Centre for Child Studies Department of Health Research Methods, Evidence and Impact (formerly Clinical Epidemiology and Biostatistics) McMaster University Hamilton, ON Canada; ^5^ Department of Community Health and Epidemiology Dalhousie University Halifax, NS Canada

**Keywords:** eHealth, mental health, implementation science, healthcare planning, organizational innovation, decision-making, healthcare organizations

## Abstract

**Background:**

Anxiety disorders and depression are frequent conditions in childhood and adolescence. eMental healthcare technologies may improve access to services, but their uptake within health systems is limited.

**Objective:**

The objective of this review was to examine and describe how the implementation of eMental healthcare technologies for anxiety disorders and depression in children and adolescents has been studied.

**Methods:**

We conducted a search of 5 electronic databases and gray literature. Eligible studies were those that assessed an eMental healthcare technology for treating or preventing anxiety or depression, included children or adolescents (<18 years), or their parents or healthcare providers and reported findings on technology implementation. The methodological quality of studies was evaluated using the Mixed Methods Appraisal Tool. Outcomes of interest were based on 8 implementation outcomes: acceptability (satisfaction with a technology), adoption (technology uptake and utilization), appropriateness (“fitness for purpose”), cost (financial impact of technology implementation), feasibility (extent to which a technology was successfully used), fidelity (implementation as intended), penetration (“spread” or “reach” of the technology), and sustainability (maintenance or integration of a technology within a healthcare service). For extracted implementation outcome data, we coded favorable ratings on measurement scales as “positive results” and unfavorable ratings on measurement scales as “negative results.” Those studies that reported both positive and negative findings were coded as having “mixed results.”

**Results:**

A total of 46 studies met the inclusion criteria, the majority of which were rated as very good to excellent in methodological quality. These studies investigated eMental healthcare technologies for anxiety (n=23), depression (n=18), or both anxiety and depression (n=5). Studies of technologies for anxiety evaluated the following: (1) acceptability (78%) reported high levels of satisfaction, (2) adoption (43%) commonly reported positive results, and (3) feasibility (43%) reported mixed results. Studies of technologies for depression evaluated the following: (1) appropriateness (56%) reported moderate helpfulness and (2) acceptability (50%) described a mix of both positive and negative findings. Studies of technologies designed to aid anxiety and depression commonly reported mixed experiences with acceptability and adoption and positive findings for appropriateness of the technologies for treatment. Across all studies, cost, fidelity, and penetration and sustainability were the least measured implementation outcomes.

**Conclusions:**

Acceptability of eMental healthcare technology is high among users and is the most commonly investigated implementation outcome. Perceptions of the appropriateness and adoption of eMental healthcare technology were varied. Implementation research that identifies, evaluates, and reports on costs, sustainability, and fidelity to clinical guidelines is crucial for making high-quality eMental healthcare available to children and adolescents.

## Introduction

Worldwide, at least 6.5% and 2.6% of children and adolescents meet the criteria for anxiety and depressive disorders, respectively [1]. The burden associated with these disorders rises sharply in childhood and peaks in adolescence and young adulthood (ages, 15-24 years) [2]. The long-term impact of anxiety and depression on children and adolescents includes significant interference with relationships, academic performance, school attendance, and daily functioning, making early intervention vital [3-8].

Underdiagnosis and undertreatment of childhood and adolescent depression and anxiety are well-documented concerns [9,10]. The current distribution, demand, structure, and costs that underpin services for these young people make them relatively unavailable to many who need them [11]. Electronic mental (eMental) healthcare technologies, which include internet-, mobile-, and, computer-based programs as well as mobile phone apps, supposedly improve mental healthcare access and availability [12-17]. In the past 5 years, a number of literature reviews have highlighted the increase in research and development activities for eMental healthcare technologies for children and adolescents [18-22]. While conclusions regarding the efficacy and effectiveness of technologies vary depending on the review and employed methodology, reviews are unified in their assessment that eMental healthcare technologies have potential utility in healthcare systems. However, despite increased emphasis on the potential value for improving health outcomes for children and adolescents, eMental health technologies are not widely adopted within health systems [23-26].

Distinguishing implementation effectiveness from the viewpoint of treatment effectiveness is critical for integrating eMental healthcare technologies. When uptake efforts fail, it is important to know if the failure occurred because the intervention was ineffective in the new setting (eg, lacked cultural relevance), or if an effective intervention was deployed ineffectively (eg, clinicians failed to send reminder emails as the protocol indicated). Current research on eMental healthcare technologies lack implementation frameworks [27], and the implementation literature has traditionally focused on the broadly defined eHealth [28,29], lacking a specific focus on mental healthcare. Conceptualizing and assessing implementation outcomes (ie, how implementation of a program works in specifics contexts) can advance the understanding of implementation processes (eg, cost, required in-service training, required infrastructure), enable studies of the comparative effectiveness of implementation strategies, and enhance efficiency in translating research into practice. The aim of this systematic review was to examine how the implementation of eMental healthcare technologies for children and adolescents with anxiety or depression has been studied (ie, the research questions asked, populations studied, and the rigor of the methodology used) and to describe implementation findings with respect to implementation processes and outcomes.

## Methods

### Design

A protocol for the review was developed and registered with PROSPERO (Registration #CRD42016049884). Reporting of the review adheres to the Preferred Reporting Items of Systematic Reviews and Meta-Analyses statement checklist [30]. Funding for the review was provided by the Canadian Institutes of Health Research (201404KRS). This organization had no involvement in any aspect of the conduct, analysis, and manuscript preparation of this review. This systematic review did not require ethics approval nor does it contain any individual person’s data in any form.

### Search Strategy

A research librarian developed the search strategies for 5 databases: MEDLINE, EMBASE, PsycINFO, CINAHL, and the Cochrane Database of Systematic Reviews using date (2000-2016) restrictions. No restriction was placed on the study design or language to capture a broad range of evidence. The strategy was peer reviewed prior to implementation. The searches included literature published until December 5, 2016. Grey literature was searched using Google Scholar and ProQuest Dissertations & Theses Global. Clinical trials were searched using clinicialtrials.gov. Conference proceedings of the last 2 years (2014-2016) of the International Society for Research on Internet Interventions were searched as well. Reference lists of included studies were also searched. Multimedia Appendix 1 provides the search terms developed for the MEDLINE database.

### Criteria for Considering Studies in the Review

Studies were included if they met the following criteria: (1) assessed an eMental healthcare technology for treating or preventing anxiety or depression; (2) the technology under investigation involved children or adolescents (<18 years), or their parents or healthcare providers. Studies that included both adolescents <18 and young adults were included if the mean age of the study sample was ≤19 years to ensure that the results largely reflected implementation with children and adolescents; (3) the technology needed to be an internet-, computer-, tablet-, or mobile-based program or mobile app; (4) the technology was used within the primary or secondary healthcare system (as opposed to the school system); (5) reported on an implementation outcome as a primary or secondary measure. The 8 outcomes of interest were drawn from Proctor and colleagues’ implementation framework [31]. These constructs were defined as follows: *acceptability* (ie, a measure of satisfaction with a technology including attitudes, functionality, preferences, and user experience); *adoption* (ie, the intention, initial decision, or action to take up or utilize a technology); *appropriateness* (ie, the perceived fit, relevance, usefulness/helpfulness, or compatibility of a technology for a given practice setting or problem); *cost*, (ie, the financial impact of an implementation effort); *feasibility*, (ie, the extent to which a technology had utility and compatibility within the practice setting); *fidelity*, (ie, the degree to which a technology was implemented as it was intended); *penetration*, (ie, the spread and reach of a technology within a service setting and its subsystems); and *sustainability*, (ie, the extent to which a technology was maintained within standard operations) [31]. We excluded protocols, editorials, and studies assessing telehealth interventions, including telepsychiatry and videoconferencing.

### Screening for Eligibility

References were organized and screened using EndNote X7.2.1. Three reviewers (AS, NDG, and MO) independently screened the titles and abstracts in the EndNote library and calculated the interrater agreement with the kappa statistic for every 100 articles screened [32]. Once a sufficiently high kappa was reached (≥0.80), the remaining references in the library were divided into 3 equally sized groups. Each reviewer was given 2 of the 3 groups, allowing each article to be assessed by 2 reviewers, and each reviewer screened the studies using the title and abstract. Three reviewers (AS, NDG, MO) independently reviewed the full-text of studies that were identified as potentially eligible using the review’s inclusion and exclusion criteria. Any discrepancies were discussed among the reviewers and taken to a third party (ASN) if no agreement could be reached.

### Data Extraction

Data were extracted by one reviewer (AS, NDG, or MO), and reviewed for accuracy and completeness by another. After verifying all of the extracted data, discrepancies were resolved by discussion or adjudication by another party (ASN). Extracted data included information on study characteristics (eg, authors, date of publication, country, and design) and implementation objectives, characteristics of the technology, study population, study setting, and implementation results. We coded statistically significant favorable ratings on measurement scales as “positive results” (eg, healthcare providers rating an intervention as highly acceptable) and statistically significant unfavorable ratings on measurement scales as “negative results” (eg, parents did not think the activities in the program were acceptable for their child’s age). Those studies that reported both positive and negative findings were coded as having “mixed results” (eg, child and parents did not show the same level of satisfaction with the intervention).

### Quality Assessment

Methodological quality was assessed independently by 2 of the 3 assessors (AS, NDG, and MO). Disagreements were resolved through discussion. ASN participated when consensus could not be reached. The quality of studies was assessed using the Mixed Methods Appraisal Tool (MMAT) [33]. The scoring scale ranges from 0 (low quality) to 100 (high quality) and was pilot tested for reliability [34]. The MMAT consists of 2 screening questions applicable to all types of study designs and 3-4 questions applicable to specific study designs (eg, The questions relevant to each study design were scored with the number of ‘yes’ answers summed, divided by the total number of questions, and multiplied by 100 to give a final percentage score.) Qualitative studies were appraised for the relevance of data sources, processes used for data analyses, consideration of study context, and the researchers’ potential influences. Randomized controlled trials (RCTs) were appraised for sequence generation, allocation concealment, the completeness of outcome data, and study attrition. All other quantitative studies were appraised for recruitment strategies and sample representativeness, outcome measurements, the completeness of outcome data and study response rates, and the comparability of comparison groups (when applicable). Mixed methods studies were assessed for the relevance of the design, integration of methods, and limitations to integration. We did not exclude any studies on the basis of low-quality assessment scores.

### Data Analysis

A codebook approach [35] was used to organize data extraction according to the 8 implementation outcome categories [31]. When no implementation data were available for a particular outcome in the included paper, the category remained empty. Four team members (NDG, MO, AS, and ASN) reviewed the assignments of the study outcome data to the implementation categories, and assignments were finalized after all team members were confident that the data were categorized accurately. Descriptive statistics (counts, frequencies) were used to summarize patterns across studies.

## Results

### Literature Search and Selection

**Figure 1:**
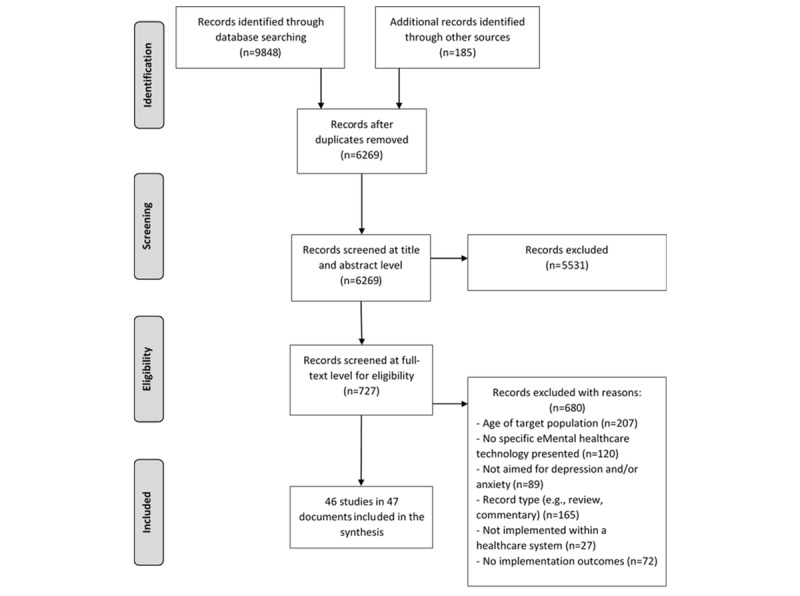
Literature search flow diagram.

The search strategy identified 6269 citations after removal of duplicates. Of these, 727 studies were considered potentially relevant based on their title and abstract (Figure 1). After full-text review, 46 studies (plus one erratum) articles met the inclusion criteria.

### Description of Included Studies

Table 1 outlines the format and delivery characteristics of the technologies assessed in the included studies. The implementation of eMental healthcare technologies for anxiety and depressive disorders in childhood or adolescence was assessed in 23 and 18 studies, respectively. Five studies assessed a technology that targeted both anxiety and depression. The location of studies was restricted to economically developed countries with the United States (20 studies) and Australia (13 studies) being the most common locations. A total of 32 studies examined internet-based technologies, 11 examined computer-based technologies, and 3 examined smartphone-based (app/short message service, SMS, text message) technologies as part of treatment.

### Study Quality

Details on the quality of the studies are provided in Multimedia Appendix 2. In total, 11 studies on eMental healthcare technologies for anxiety were of excellent quality with a score of 100 [37,46-50,52,55,78-80], 6 were of very good quality with a score of 75 [36,38,39,44,51,53], 4 were of moderate quality with a score of 50 [42,43,45,54], and 2 were of poor quality with a MMAT score of 25 [40,41]. Studies on technologies for depression also varied in quality: 10 studies were of excellent quality [56,57,59,63-67,71,81], 2 were of very good quality [60,68], 4 were of moderate quality [58,61,69,72], and 2 were of low quality and received a score of 25 [62] and 0 [70]. Studies evaluating technologies applicable to both anxiety and depression were of excellent [75,77], very good [74], and moderate [73,76] quality. The most common factors impacting the quality scores for quantitative studies were the lack of description on how randomization sequences were generated and if/how allocation was concealed (ie, see MMAT items 2.1 and 2.2 in Multimedia Appendix 2). The common factor impacting quality scores for mixed-method studies was the lack of consideration of data triangulation (ie, see MMAT item 5.3 in Multimedia Appendix 2).

**Table 1:** Reported format and delivery characteristics of eMental Health technologies for adolescents with anxiety and depression.

Technology/Program Name		Participants	Technology Details			
		Target age (years)	Parent involvement	Features (sessions)	Healthcare provider contact	
					Before program	During program
**Anxiety Programs**						
	Cool Little Kids Online [36]	3-6	Yes	Internet-based (8 modules)	None	Phone
	Camp-Cope-A-Lot [37-41]	7-13	Yes	Computer-based (12 sessions)	In-person	In-person^a^
	DARE Program [42,79]	8-12	Yes	Internet-based (11 modules)	None	Phone, within program^b^
	BiP OCD [78,80]	12-17	Yes	Internet-based (12 chapters)	None	Within program
	BRAVE-ONLINE^c^ [46-50]	7-18^d^	Yes	Internet-based (10 sessions)	None	Email, within program
	Cognitive bias modification [43]	10-15	Yes	Internet-based (8 sessions)	In-person^e^	None
	Ricky and the Spider [51]	6-12	Yes	Internet-based (8 levels)	In-person	In-person
	Cool Teens [44,52,53]	14-18	No	Computer-based (8 modules)	None	Phone
	Self-help manual and treatment [54]	15-21	No	Internet-based (9 modules)	None	Within program
	SmartCAT App [45]	9-14	No	Mobile-based app (Ad hoc; includes 5 main components)	None	In-person, within program
	Virtual School Environment [55]	8-12	Yes	Computer-based (12 sessions)	In-person	In-person
**Depression Programs**						
	Decision aid tool [56]	12-25	No	Internet-based (9 component webpage used during an appointment or in the waiting room)	None	In-person
	Monitoring tool [57,58]	15-24	No	Internet/tablet-based (Depression assessments)	None	In-person^f^
	Rebound (Australia) [59]	15-25	No	Internet-based (User can select from 56 sessions)	None	Within program
	MAYA (Chile) [81]	12-18	No	Internet-based (1 session)	In-person	In-person
	iDOVE (United States) [60]	13-17	No	Mobile-based (8 weeks of 2 way SMS^g^ text messaging)	In-person	SMS text message
	Technology-enhanced CBT^h^ intervention (United States) [61]	12-17	No	Mobile/tablet-based (SMS text messaging)	None	In-person, SMS text message
	Behavioral Activation (United States) [62]	12-17	No	Internet-based (Ad hoc)	None	None
	CATCH-IT (United States) [63-70]	14-21	Yes	Internet-based (11-14 modules)	In-person	Phone
	SPARX (Australia) [71]	12-19	No	Computer-based (7 modules)	None^h^	Phone
	Depression Experience Journal (United States) [72]	8-19	Yes	Internet-based (Ad hoc)	In-person	None^e^
**Anxiety + Depression Programs**						
	Multi-family group therapy (Canada) [73]	6-12	Yes	Internet-based (3 sessions)	None	Email
	Treasure Hunt (Switzerland) [74]	9-13	No	Internet-based (6 levels)	None	In-person
	SPARX (New Zealand) [75]	16-18	No	Computer-based (7 modules)	None	In-person
	Problem-solving therapy (Netherlands) [76]	12-21	No	Internet-based (5 lessons)	None	Within program
	RU-OK (United Kingdom) [77]	13-15	No	Internet-based (Ad hoc)	None	None^e^

^a^Sessions 1-6 were self-led, but conducted in the presence of a healthcare provider; sessions 7-12 were primarily led by a healthcare provider.

^b^Within program refers to communication self-contained within the program (internal email program). In this case, the user would have to login to see the communication that would not be delivered to their external email.

^c^Intervention has been modified for different age groups under slightly different names.

^d^BRAVE for children-ONLINE targets participants aged 7-14 years; BRAVE for teenagers-ONLINE targets participants aged 12-18 years.

^e^Intervention did not contain healthcare provider contact, but participants were referred by healthcare providers or were engaged with the healthcare system.

^f^Participants did not use the intervention for healthcare provider interaction; providers received data or email updates that were used in in-person sessions.

^g^SMS: short message service.

^h^CBT: cognitive behavioral therapy.

^i^SPARX was tested in different implementation contexts, some of which included no in-person contact and some with in-person contact.

### Trends in the Study of Implementation Among eMental Healthcare Technologies

**Figure 2:**
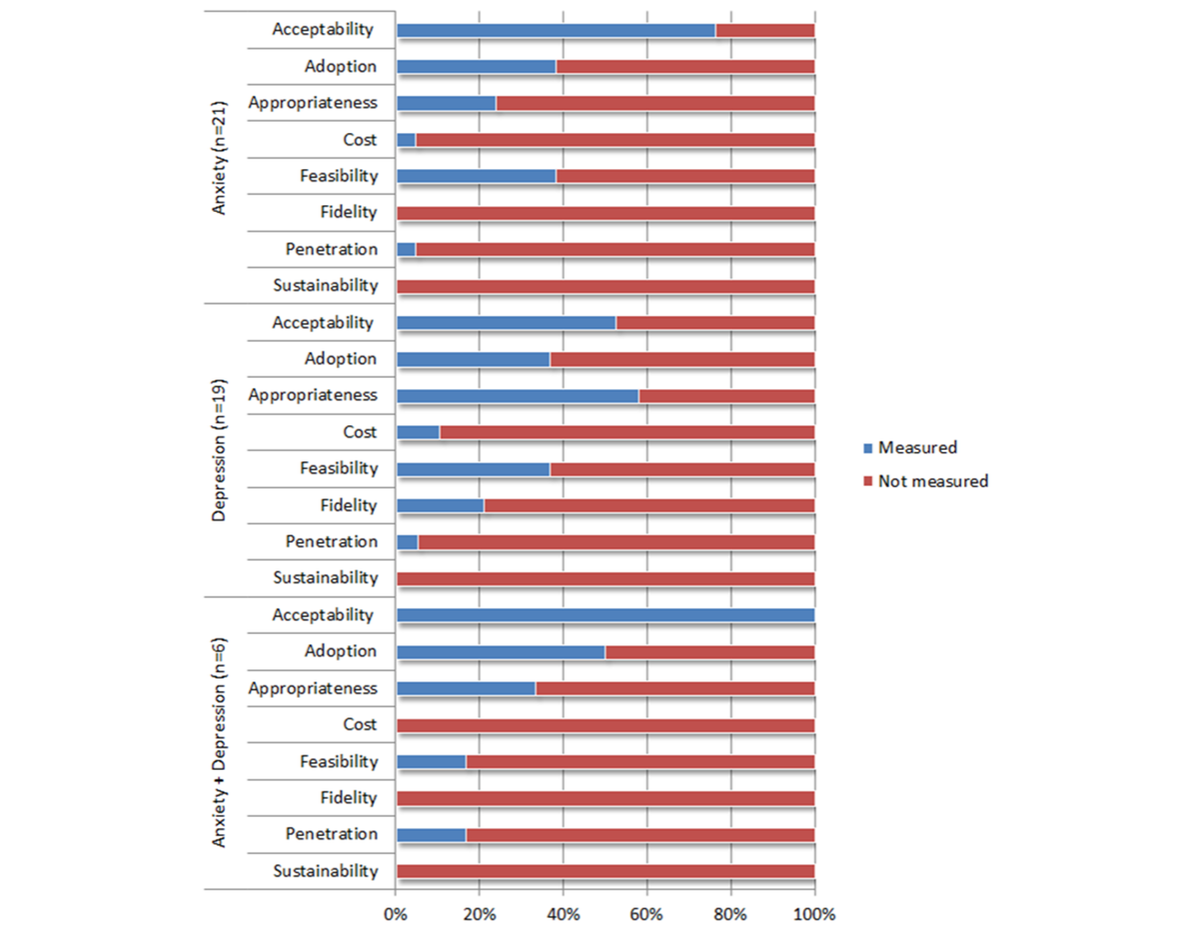
Implementation outcomes measured according to the mental health condition targeted.

**Figure 3:**
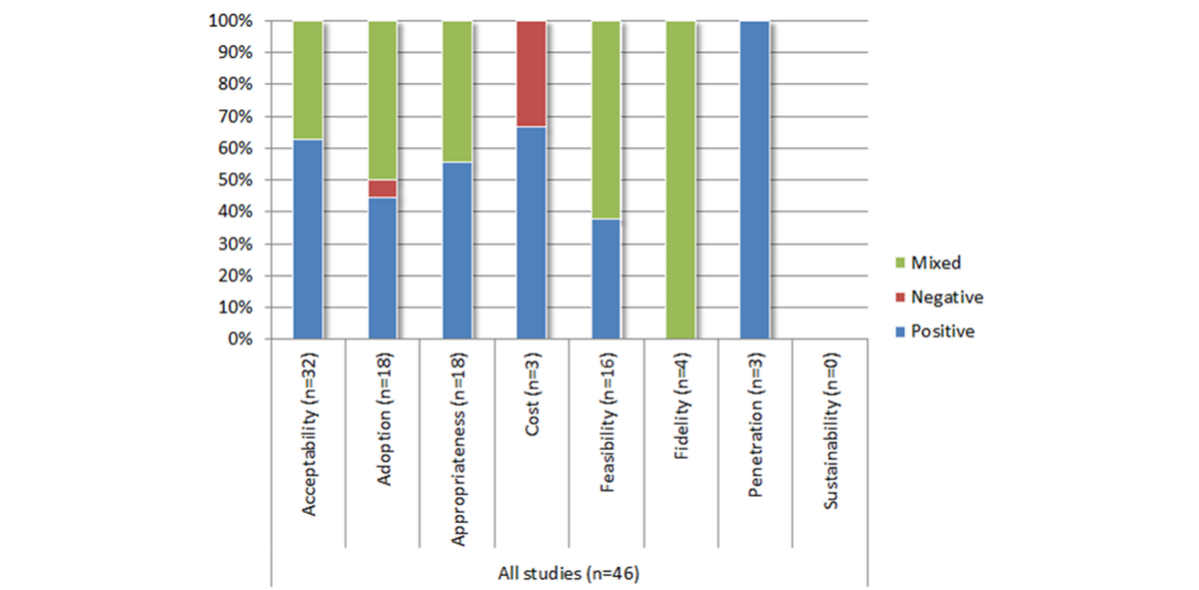
Conclusions reported by the authors for implementation outcomes.

Figure 2 displays the frequency by which implementation outcomes were studied for eMental healthcare technologies. Studies on eMental healthcare technologies for anxiety most commonly evaluated acceptability (78%), adoption (43%), and feasibility (43%) of the technologies, while studies on technologies for depression evaluated appropriateness (56%) and acceptability (50%). Studies testing technologies relevant to both anxiety and depression tended to evaluate acceptability (100%), adoption (40%), and appropriateness (40%). Across all studies, cost, fidelity, and penetration were the least measured implementation outcomes, and none of the studies evaluated technology sustainability in the healthcare service/system in which the technology was employed. While positive findings were reported 60% of the time or more in relation to measures of acceptability and costs across all included studies (Figure 3), mixed findings were reported more than 50% of the time in studies that measured adoption, feasibility, and fidelity outcomes.

### Implementation Findings for eMental Healthcare Technologies for Anxiety

Table 2 outlines the implementation findings among eMental healthcare technologies for anxiety. Both positive (61%) [36,38,39,41,43,45,50,54,55,78,80] and mixed (39%) [37,40,42,46,48,49,79] findings were reported across 18 studies on technology acceptability. Positive results included high satisfaction and positive technology recommendations, with acceptability reported by parents [36,39,41,43,50], children [38,39,41,43,45,50,54,55,78,80], and healthcare providers [55]. Technology adoption was examined by 10 studies with studies reporting positive (60%) [42-44,47,50,53] and mixed (40%) [45,46,55,79] findings for technology compliance and adherence. Of the 6 studies that examined appropriateness, 4 described positive results (67%) [39,50,51,78] such as positive attitudes and perceived helpfulness of the technology among healthcare providers [39,51], while 2 studies [53,55] reported mixed results (33%) including moderate usefulness and helpfulness of the program for the youth [53]. Of the 23 studies on anxiety-directed technologies, only one examined cost, including initial implementation challenges such as startup costs, designated computers and clinic space, and technical assistance requirement [39]. Studies that examined the feasibility of anxiety technologies described more mixed (70%) [38-40,44,52,53,55] than positive (30%) [36,45,80] results, including barriers to participation such as finding time to complete tasks and ease of use. Only one study investigated technology penetration, reporting positive penetration with technology purchased by 56 child psychiatric institutions or practitioners within 1 year [51]. Studies examining eMental healthcare technologies for anxiety did not investigate or report on fidelity or sustainability.

**Table 2:** Implementation findings among eMental healthcare technologies for anxiety.

Program and study		Participants (n)	Implementation outcome (measure^a^); findings^b^
**Cool Little Kids Online**			
	Morgan et al [36]	Parents of children aged 3-6 years with anxiety problems (n=51)	Acceptability (self-developed questionnaire); P: + Feasibility (self-developed questionnaire); P: +
**Camp-Cope-A-Lot**			
	Salloum et al [40]	Parents of children aged 7-13 years with an anxiety disorder (n=100)	Acceptability (published instrument); P: +/– Feasibility (published instrument); P: +/–
	Storch et al [41]	Children aged 7-13 years with an anxiety disorder (n=49)	Acceptability (published instrument); C: +
	Salloum et al [39]	Children aged 7-13 years with an anxiety disorder (n=3) and their parents (n=7) Healthcare providers (n=3) Project coordinators (n=3) Administrators (n=3)	Acceptability (published instrument); P, C: + Appropriateness (self-developed interview); HCP: + Cost (self-developed interview); HCP, A: – Feasibility (published instrument & self-developed interview); HCP, A, PC: +/–
	Crawford et al [37]	Children aged 7-13 years with an anxiety disorder (n=17)	Acceptability (published instrument); C: +/–
	Khanna and Kendall [38]	Children aged 7-13 years with an anxiety disorder (n=16)	Acceptability (published instrument); C: + Feasibility (self-developed questionnaire); C: +/–
**DARE Program**			
	Vigerland et al [42]	Children (n=46) aged 8-12 years with an anxiety disorder and their parents (n=46)	Acceptability (published instrument); P, C: +/– Adoption (program utilization); P, C: +
	Vigerland et al [79]	Children aged 8-12 years with social phobia (n=30) and their parents (n=57)	Acceptability (published instrument); C: + Adoption (program utilization); C: +/–
**BiP OCD^c^**			
	Lenhard et al [80]	Adolescents aged 12-13 years with OCD (n=8)	Acceptability (self-developed interview); C: + Feasibility (self-developed interview); C: +
	Lenhard et al [78]	Adolescents aged 12-17 years with OCD (n=21)	Acceptability (self-developed questionnaire); C: + Appropriateness (self-developed questionnaire); C: +
**BRAVE ONLINE**			
	Donovan and March [46]	Children aged 3-6 with an anxiety disorder (n=23)	Acceptability (self-developed questionnaire); C: +/– Adoption (program utilization); C: +/–
	Anderson et al [47]	Children and adolescents aged 7-18 years with an anxiety disorder (n=132) and their parents (n=NR^d^)	Adoption (program utilization); P, C: +
	Spence et al [48]	Adolescents aged 12-18 years with clinical levels of anxiety (n=44)	Acceptability (adapted questionnaire); C: +/–
	March et al [49]	Children aged 7-12 years with an anxiety disorder (n=40) and their parents (n=NR)	Acceptability (self-developed questionnaire); P, C: +/–
	Spence et al [50]	Children and adolescents aged 7-14 years with clinical levels of anxiety (n=27) and their parents (n=NR)	Acceptability (self-developed questionnaire); P, C: + Adoption (program utilization); P, C: + Appropriateness (self-developed questionnaire); P: +
**Cognitive bias modification**			
	Reuland and Teachman [43]	Children and adolescents aged 10-15 years with social anxiety and their mothers (n=18 mother-child dyads)	Acceptability (self-developed interview); P, C: + Adoption (program utilization); P, C: +
**Ricky and the Spider**			
	Brezinka [51]	Children and adolescents aged 6-13 years with OCD (n=18) Healthcare providers (n=13)	Appropriateness (self-developed questionnaire); HCP: + Penetration (uptake by practices); HCP: +
**Cool Teens**			
	Wuthrich et al [53]	Adolescents aged 14-17 years with an anxiety disorder (n=24)	Adoption (program utilization); C: + Appropriateness (self-developed questionnaire); C: +/– Feasibility (self-developed questionnaire); C: +/–
	Cunningham et al [52]	Adolescents aged 14-18 years with an anxiety disorder (n=22) Nonclinical adolescents (n=13)	Feasibility (self-developed questionnaire); C: +/–
	Cunningham and Wuthrich [44]	Adolescents aged 14-16 years with an anxiety disorder (n=5)	Adoption (program utilization); C: + Feasibility (self-developed questionnaire); C: +/–
**Virtual School Environment**			
	Sarver et al [55]	Children aged 8-12 years with a principal diagnosis of social anxiety disorder (n=17) Healthcare providers (n=NR)	Acceptability (self-developed questionnaire); C, HCP: + Adoption (program utilization); C: +/– Appropriateness (self-developed questionnaire); C: +/– Feasibility (successful use & technical difficulties); C, HCP: +/–
**SmartCAT App**			
	Pramana et al [45]	Children and adolescents aged 9-14 years with a diagnosis of GAD^e^, social or specific phobia, attention deficit hyperactivity disorder, oppositional defiant disorder, or social anxiety disorder (n=9)	Acceptability (self-developed questionnaire); C: + Adoption (program utilization); C: +/– Feasibility (published instrument); C: +
**Self-help**			
	Tillfors et al [54]	Adolescents aged 15-21 years with social anxiety disorder (n=10)	Acceptability (self-developed questionnaire); C: +

^a^Self-developed questionnaire/interview: bespoke questions or survey items created by the researcher; published instrument: validated tool with citation in text; program utilization/physician adherence: metrics of usage.

^b^C: child/adolescent/young adult report; HCP: healthcare provider report; P: parent report; +: high/positive findings; – negative findings; +/– mixed findings.

^c^OCD: obsessive-compulsive disorder.

^d^NR: not reported.

^e^GAD: Generalized anxiety disorder.

**Table 3:** Implementation findings among eMental healthcare technologies for depression.

Program and study			Participants (n)		Implementation outcome (measure^a^); findings^b^
**SPARX**					
	Merry et al [71]		Adolescents aged 12-19 years with depressive symptoms (n=94)	Acceptability (self-developed questionnaire); C: +	
**Depression Experience Journal**					
	Demaso et al [72]		Primary caregivers (n=38) of hospitalized adolescents aged 8-19 years	Acceptability (self-developed interview); P: + Appropriateness (self-developed interview); P: +/–	
**Behavioral activation intervention**					
	Davidson et al [62]		Adolescents aged 12-17 years with clinical and subclinical depression (n=24)	Appropriateness (self-developed questionnaire); C: + Feasibility (voiced opinions); C: + Fidelity (voiced opinions); C: +/–	
**CBT^c^**					
	Kobak et al [61]		Adolescents aged 12-17 years with clinical and subclinical depression (n=24)	Acceptability (published instrument); C, HCP: + Appropriateness (self-developed questionnaire); HCP: +	
**Decision aid**					
	Simmons et al [56]		Adolescents and young adults aged 12-25 years with mild to moderate-severe depression (n=66)	Acceptability (published instrument); C: + Adoption (program utilization); C: +/–	
**Monitoring tool**					
	Hetrick et al [58]		Adolescents and young adults aged 14-25 years diagnosed with depressive symptoms or a depressive disorder (n=101) FHealthcare providers (n=33)	Acceptability (self-developed questionnaire); C, HCP: + Appropriateness (self-developed questionnaire); C, HCP: +/–	
	Hetrick et al [57]		Adolescents and young adults aged 15-25 years diagnosed with major depressive disorder (n=15) FHealthcare providers (n=7)	Appropriateness (self-developed questionnaire & interview); C, HCP: + Feasibility (self-developed interview); C, HCP: +/–	
**Rebound**					
	Rice et al [59]		Adolescents and young adults aged 15-24 years in partial or full remission of major depressive disorder (n=42)	Adoption (program utilization); C: +	
**MAYA**					
	Carrasco [81]		Female adolescents aged 12-18 years with symptoms of depression (n=15) Healthcare providers (n=5)	Acceptability (self-developed questionnaire); C: +/– Feasibility (self-developed questionnaire); C: +/–	
**iDOVE**					
	Ranney et al [60]		Adolescents aged 13-17 years at high risk for depression and with a past-year history of physical peer violence (n=16)	Acceptability (adapted published instrument); C: +/– Adoption (program utilization); C: +/– Appropriateness (self-developed interview); C: + Feasibility (adapted published instrument); C: +	
**CATCH-IT**					
	Gladstone et al [69]		Adolescents and young adults aged 14-21 years with subthreshold depression (n=83)	Appropriateness (adapted questionnaire); C: +/– Feasibility (adapted questionnaire); C: +	
	Ruby et al [67]		Adolescents and young adults aged 14-21 years with subthreshold depression (n=83)	Cost (economic analysis); C: +	
	Eisen et al [70]		Adolescents and young adults aged 14-21 years with subthreshold depression (n=83) Healthcare providers (n=63)	Acceptability (self-developed questionnaire); HCP: +/– Appropriateness (self-developed questionnaire); HCP: +/– Feasibility (self-developed questionnaire); HCP: +/–	
	Iloabachie et al [68]		Adolescents and young adults aged 14-21 years with subthreshold depression (n=83)	Appropriateness (adapted questionnaire); C: +/–	
	Van Voorhes et al [66]		Adolescents with subthreshold depression (n=83) Primary healthcare providers (n=12) Healthcare settings (n=5)	Cost (marketing strategy success/cost reporting); HCP: + Penetration (uptake by practices); HCP: +	
	Van Voorhes et al [63]		Adolescents and young adults aged 14-21 years with subthreshold depression (n=83)	Acceptability (self-developed questionnaire); C: + Adoption (program utilization); C: +/– Appropriateness (self-developed questionnaire); C: +/– Fidelity (physician adherence); HCP: +/–	
	Van Voorhes et al [64,65]		Adolescents and young adults aged 14-21 years with subthreshold depression (n=83)	Adoption (program utilization); C: +/– Fidelity (physician adherence); HCP: +/–	


^a^Self-developed questionnaire/interview: bespoke questions or survey items created by the researcher; published instrument: validated tool with citation in text; program utilization/physician adherence: metrics of usage.

^b^C: child/adolescent/young adult report; HCP: healthcare provider report; P: parent report; +: high/positive findings; – negative findings; +/– mixed findings.

^c^CBT: cognitive behavioral therapy.

### Implementation Findings for eMental Healthcare Technologies for Depression

Table 3 displays the implementation findings among eMental healthcare technologies for depression. Most studies reported the technologies as acceptable (67%) with high satisfaction [56,61,63,72], recommendations for use [71], acceptability, and ease of use among children, parents, and healthcare providers [58]. The remainder (33%) reported mixed acceptability [60,70,81]. Of the 6 studies that examined adoption, one study (17%) described high usage [59], while the remaining studies (83%) described moderate or mixed adherence [56,63-65] and usage [60]. Appropriateness was the most commonly measured outcome among eMental healthcare technologies for depression, although results varied. Four studies (40%) reported high helpfulness [57,60-62], while 6 studies (60%) reported mixed outcomes [58,63,68-70,72]. Two studies examined cost outcomes [66,67] and described intervention implementation as economically viable. Of the 6 studies that investigated feasibility, 3 (50%) reported positive or high outcomes [60,62,69], while the other 3 (50%) described mixed ease of use [70,81] and attitudes [57]. Four studies examining fidelity reported mixed results [62-65], particularly healthcare provider adherence to the program. The CATCH-IT program was the only intervention that was examined for penetration [66]. Although penetration was successful, implementing the technology successfully in 12 practices, several barriers to implementation were described, such as low levels of interest from healthcare providers and lack of established procedures and guidelines [66]. Studies examining eMental healthcare technologies for depression did not investigate or report on sustainability.

### Implementation Findings for eMental Healthcare Technologies for Anxiety and Depression

Table 4 shows the implementation findings among eMental healthcare technologies for both anxiety and depression. All 5 studies examined the acceptability of technologies aimed at treating anxiety and depression. Of these, 3 (60%) reported high satisfaction [73-75], with children and parents describing that they would not change any aspects of the program, and 2 studies (40%) reported moderate satisfaction [76,77]. Two studies examined adoption and reported low adherence to program sessions [75] and high website usage rates [77]. Of the 2 studies that examined appropriateness, both found positive attitudes and perceived helpfulness of the intervention from children and parents [73] and healthcare providers [74]. One study examined penetration of technology, reporting successful integration of the program into a practice of 2000 healthcare providers [74]. None of the 5 studies examined cost, feasibility, fidelity, or sustainability.

**Table 4:** Implementation findings among eMental healthcare technologies for both anxiety and depression.

Program and study		Participants (n)	Implementation outcome (measure^a^); findings^b^
**Group therapy**			
	Sapru et al [73]	Children aged 6-12 years referred with a mood or anxiety disorder (n=16) and their parents (n=NR^g^)	Acceptability (self-developed questionnaire); C, P: + Appropriateness (open-ended feedback); C, P: +
**Treasure Hunt**			
	Brezinka [74]	Children and adolescents aged 6-19 years with anxiety, depression, ODD^c^, or ADHD^d^ (n=218) Healthcare providers (n=124)	Acceptability (self-developed questionnaire); C: + Appropriateness (self-developed questionnaire); HCP: + Penetration (uptake by practices); HCP: +
**SPARX**			
	Bobier et al [75]	Adolescents aged 16-18 years with severe psychiatric disorders (namely mood and anxiety disorders; n=20)	Acceptability (self-developed questionnaire); C: + Adoption (program utilization); C: –
**Problem-solving therapy**			
	Hoek et al [76]	Adolescents and young adults aged 12-21 years with self-reported or parent-reported mild to moderate depressive or anxiety symptoms (n=22)	Acceptability (published instrument); C: +/–
**RU-OK**			
	Ercan et al [77]	Adolescents aged 13-15 years attending a hospital school for depression and anxiety (n=105)	Acceptability (self-developed questionnaire); C: +/– Adoption (program utilization); C: +

^a^Self-developed questionnaire/interview: bespoke questions or survey items created by the researcher; published instrument: validated tool with citation in text; program utilization/physician adherence: metrics of usage.

^b^C: child/adolescent/young adult report; HCP: healthcare provider report; P: parent report; +: high/positive findings; – negative findings; +/– mixed findings.

^c^NR: not reported.

^d^ODD: oppositional defiant disorder.

^e^ADHD: attention deficit hyperactivity disorders.

## Discussion

### Principal Findings

Complimentary to recent reviews [12,82], this systematic review reports on how the implementation of eMental healthcare technologies for children and adolescents with anxiety or depression has been studied and reported. The majority of studies included in the review were RCTs, and the methodological quality of studies was scored as moderate to high in all but a few cases. Broadly synthesized using Proctor’s [31] 8 dimensions of implementation, our review suggests that measures of acceptability, adoption, and appropriateness are more frequently reported than indicators of cost, fidelity, and sustainability. Further, the review highlights the lack of measurement precision around implementation constructs and the need to elucidate the relationship between implementation and effectiveness. Below, we highlight 5 key implications of our findings for advancing this emerging literature. Results derived from new lines of research can have significant practical value for decision-makers and administrators by providing the design of training, helping promote provider engagement, assisting in troubleshooting the obstacles that adolescents and parents encounter, and guiding projects that scale-up interventions in new contexts.

### Improving the Validity of Acceptability Measures

The vast majority of studies included in the review examined some dimension of acceptability, signifying that this construct is important as an indicator of effective implementation, but its measurement varied. Satisfaction, a frequent acceptability metric, was reported as high among participants (generally >70%), but was largely derived from self-reports of parents and adolescents taken at a single time-point (typically posttreatment). This means we still know little about satisfaction/dissatisfaction among those who fail to complete the treatment, or how early perceptions of satisfaction might impact effort and adherence during the later stages of treatment. More than half of the studies used nonvalidated measures of acceptability, which are problematic for assessing reliability and psychometric sensitivity. Given that almost all validated psychiatric patient satisfaction measures are validated for adults (not children and adolescents) and that developmental age affects perceptions of satisfaction with healthcare [83], our findings raise the possibility of overestimated satisfaction ratings within this literature. Low actual adherence rates reported in many studies, particularly those treating depression [84], suggest that we need to know more about the relationship among satisfaction, adherence, and clinical improvement. Most importantly, differences between those who do and do not respond to inquiries about service satisfaction (ie, bias in nonresponse [85]) and the impact of novelty (ie, bias resulting from perceived “new” or “innovative” technology [86]) imply that satisfaction is a potentially tendentious implementation metric. Without psychometrically strong and developmentally appropriate measures of satisfaction and acceptability for eMental health, stakeholders run the risk of focusing on the wrong “pragmatic” attributes when determining if a given adolescent-focused intervention is worth long-term investment. As a metric frequently used to inform decision-making around service delivery, a more systematic approach to instrumentation around the acceptability construct is vital. Future research can use pragmatic trial designs and hybrid effectiveness-implementation designs that aim to elucidate mechanisms of action between acceptability and effectiveness.

**Reframing Adoption as Process not Product**

While reporting on adoption (namely adherence) was fairly common in studies we reviewed, authors reported mixed findings. Moreover, none of the studies in our review formatively examined adolescent, parent, or clinician adoption in terms of readiness for eMental health, intent-to-use, or ongoing decision-making. All of these factors play a central role in behavior change associated with effective mental health treatments [87,88,89]. As adoption continues to be conceptualized in the literature primarily as a posthoc measure of “adherence,” our review suggests process-related measures of adoption could be a valuable new line of research. Most of the studies in our review reported on interventions involving multiple sessions (ie, anxiety interventions had a minimum of 8 sessions), requiring the youth to sustain and repeat interactions over time. Research from other fields, like adolescent online learning and gaming, could provide important insights here. For example, research has shown that young people’s internet self-efficacy, self-regulatory skills, and perceived quality of online learner-instructor interaction are important predictors of online engagement [90]. Rather than viewing adoption as a relatively stable end-product of individual effort, emphasis should be placed on understanding the situated, mutually constitutive relationship of a young person and the eMental healthcare environment. For example, use of modeling and path analysis techniques to identify the direct and indirect effects of provider (eg, therapeutic alliance, communication style) and technical (eg, persuasive system design components) or therapeutic (eg, comorbidity, treatment credibility) factors on adoption may provide valuable and practical insights. Improved knowledge of these processes could help administrators design training, promote provider engagement, and pre-emptively address obstacles for youth and their families.

### Perceived Suitability of eMental Healthcare for Adolescents With Anxiety and Depression

A little less than half of the studies testing depression interventions and a third of those focused on anxiety measured some dimension of “appropriateness,” with many reporting overall mixed results. Perceptions about the suitability of a given healthcare service in a particular setting, for a particular purpose, with a particular provider and clientele can be a function of organizational culture and climate, as well as a public opinion. In practice, eMental healthcare is still considered outside standard practice by most youth mental health service providers [91]; yet, investments in eMental healthcare are rarely withdrawn because of purported safety risks or over concerns about the quality of care. This suggests that administrators, providers, and the general public feel that eMental healthcare is an appropriate treatment modality, but still continue to prioritize its use in some contexts over others. It may because the treatment ideologies (ie, beliefs about the etiology of illness, the roles of the provider/patient, and the efficacy of various treatments) [92] held by clinicians, parents, and children/youth lead them to greater skepticism about whether eMental healthcare can deliver the same quality of care [93] as face-to-face services for children and adolescents. In particular, public opinion and clinician beliefs about depression-associated risks (eg, suicide, self-harm [94]), privacy [95], and the changes in provider-patient interaction via eHealthcare delivery [96]) could impact perceived appropriateness. This could be one explanation for the higher acceptability rates of anxiety-focused interventions than of depression-focused ones. Research on appropriateness would benefit from an exploration of how eMental healthcare treatment ideologies develop for different clinical contexts (ie, diagnosis, severity) and technological modalities (ie, teleconsultation, mobile apps, SMS text messaging) and assess their subsequent influence on other implementation factors. These lines of research could eventually assist providers in selecting eMental healthcare technologies to match the intensity of treatment with the complexity of the condition (ie, stepped care).

### Disruption of Established Professional Roles, Responsibilities, and Working Styles

Findings from this review also make an important contribution to expanding our understanding of feasibility. The feasibility results observed in our review were most frequently related to provider-level concerns (eg, issues of training, need for technical support). This suggests that the workflow impacts of eMental health services are a vital area for future implementation research. Given that most of the eMental healthcare technologies in our review included some form of healthcare provider interaction before or during the treatment, their role cannot be underappreciated. Many of the studies described atypical interactions for providers trained in traditional psychotherapy, including use of SMS text messages, frequent short emails, and bidirectional electronic exchanges, technical support, etc. Our review echoes recent calls to move beyond the simplistic analyses of barriers and facilitators to models of feasibility that allow researchers to test how eHealth modalities disrupt established professional roles, responsibilities, and working styles [97]. We recommend increased emphasis on underdeveloped implementation outcomes like feasibility, where few comprehensive and validated instruments exist [98]. Knowledge generated from this research could inform strategic targeting of resources and the tailoring of implementation strategies at an early stage to maximize opportunities for normalization of new eMental health workflows. Studies in our review were limited by small sample sizes and were mostly focused on measuring clinician attitudes with a lesser focus on quantifying actual clinician behaviors. Policy-focused research involving clinical practice models for eMental health [99], effective training practices for eMental health, and guidelines for selecting safe and effective eMental health tools are needed to shape behaviors that will make eMental health feasible in routine care settings.

### Toward Sustainable, Cost-Effective, Scalable eMental Health for Anxiety and Depression

Finally, this review highlights persistent gaps in the measurement of fidelity, penetration, and sustainability constructs. These implementation facets are important macro-level determinants of policies and strategies for technology integration [100,101]. However, because these factors often require longer-term follow-up to adequately assess, they pose unique methodological challenges for researchers. For example, sustainability and penetration constructs typically require very large sample sizes that are hard to obtain [102] and there is concern that the current methodological approaches for eMental healthcare technology have a long lag-time from initiation, to publication of outcomes or implementation. While the promise of scalable, more cost-effective treatments is widely argued in eMental health planning, there are knowledge gaps pertaining to how these services are costed, billed, and supported in the long term. As implementation research matures in this area, it will be critical to apply research methodologies that optimize the ecological validity of constructs and address these practical, real-world implications [103]. The use of structured, theory-driven implementation methodologies would provide flexibility to allow interventions to be adapted for use in routine care settings [99,104].

### Limitations

Although this review was rigorous, carefully executed, and employed a robust methodological approach, it is not without limitations. Technologies being deployed in healthcare systems that have not been scientifically investigated and without reported implementation data were not available for our review. We did not search databases such as the NIH Reporter, which may have yielded additional eMental healthcare technology studies. While some of the studies in the NIH Reporter may have been additionally registered in the clinicaltrials.gov registry after our search, some may not have been. Given that eMental healthcare technologies are constantly appearing and disappearing from the behavioral health service landscape, published accounts of the state of this field will likely always be slightly outdated. This is true for all eHealth-related research syntheses, and it only underscores the need to promote an “evergreen” mentality for research that acknowledges that the evidence base is always evolving. The inclusion of multiple study designs created a challenge for summarizing study features and generalizing study findings. Nonetheless, this approach allowed for the comparison of different kinds of evidences that shape real-world policy and service delivery. By not limiting our search based on study design, but rather reporting on quality via a validated appraisal tool, we established a starting point for broad critical appraisal. Finally, the inconsistent use of eHealth terminology [105-107] across the literature required us to make judgment calls regarding how to group implementation data across the 8 outcome categories. This could have resulted in the misclassification of some factors within the wrong outcome category [31].

### Conclusions

Acceptability of eMental healthcare technology appears to be high among users, and it is the most commonly investigated implementation outcome. Perceptions of the appropriateness of eMental healthcare technology for use in healthcare varied, as did the adoption of technologies in healthcare practice. These findings suggest that the implementation science of eMental health for adolescent anxiety and depression needs to mature. Validated implementation measures as well as research designs and analytic techniques that model complex interactions and implementation contexts should be pursued in earnest. Future studies should help bridge gaps in knowledge about the fidelity of eMental health interventions over time and how eMental health technologies spread through the healthcare system, direct and indirect costs, and sustainability models. Closing these knowledge gaps has the potential to make treatments more accessible and reduce the burden of anxiety and depression on affected children and adolescents.

## Appendix

Multimedia Appendix 1 Search strategy for Epub ahead of print, in-process, & other non-indexed citations, Ovid MEDLINE(R) Daily and Ovid MEDLINE(R).

Multimedia Appendix 2 Study Quality according to Mixed Methods Appraisal Tool (MMAT).

## Abbreviations

MEDLINE: Medical Literature Analysis and Retrieval System Online, or MEDLARS Online
EMBASE: Excerpta Medica dataBASE
CINAHL: Cumulative Index to Nursing and Allied Health Literature
MMAT: Mixed Methods Appraisal Tool
RCT: randomized controlled trials

## References

[1] Polanczyk GV, Salum GA, Sugaya LS, Caye A, Rohde LA (2015). Annual research review: A meta-analysis of the worldwide prevalence of mental disorders in children and adolescents. J Child Psychol Psychiatry.

[2] Whiteford HA, Degenhardt L, Rehm J, Baxter AJ, Ferrari AJ, Erskine HE, Charlson FJ, Norman RE, Flaxman AD, Johns N, Burstein R, Murray CJL, Vos T (2013). Global burden of disease attributable to mental and substance use disorders: findings from the Global Burden of Disease Study 2010. Lancet.

[3] Essau CA, Lewinsohn PM, Olaya B, Seeley JR (2014). Anxiety disorders in adolescents and psychosocial outcomes at age 30. J Affect Disord.

[4] Fergusson DM, Woodward LJ (2002). Mental health, educational, and social role outcomes of adolescents with depression. Arch Gen Psychiatry.

[5] Mazzone L, Ducci F, Scoto MC, Passaniti E, D'Arrigo VG, Vitiello B (2007). The role of anxiety symptoms in school performance in a community sample of children and adolescents. BMC Public Health.

[6] Naicker K, Galambos NL, Zeng Y, Senthilselvan A, Colman I (2013). Social, demographic, and health outcomes in the 10 years following adolescent depression. J Adolesc Health.

[7] Verboom CE, Sijtsema JJ, Verhulst FC, Penninx BWJH, Ormel J (2014). Longitudinal associations between depressive problems, academic performance, and social functioning in adolescent boys and girls. Dev Psychol.

[8] Woodward LJ, Fergusson DM (2001). Life course outcomes of young people with anxiety disorders in adolescence. J Am Acad Child Adolesc Psychiatry.

[9] Merikangas KR, He J, Brody D, Fisher PW, Bourdon K, Koretz DS (2010). Prevalence and treatment of mental disorders among US children in the 2001-2004 NHANES. Pediatrics.

[10] Pelletier L, O'Donnell S, Dykxhoorn J, McRae L, Patten SB (2017). Under-diagnosis of mood disorders in Canada. Epidemiol Psychiatr Sci.

[11] Hickie IB, McGorry PD (2007). Increased access to evidence-based primary mental health care: will the implementation match the rhetoric?. Med J Aust.

[12] Boydell KM, Hodgins M, Pignatiello A, Teshima J, Edwards H, Willis D (2014). Using technology to deliver mental health services to children and youth: a scoping review. J Can Acad Child Adolesc Psychiatry.

[13] Christensen H, Hickie IB (2010). E-mental health: a new era in delivery of mental health services. Med J Aust.

[14] Christensen H, Hickie IB (2010). Using e-health applications to deliver new mental health services. Med J Aust.

[15] Hollis C, Morriss R, Martin J, Amani S, Cotton R, Denis M, Lewis S (2015). Technological innovations in mental healthcare: harnessing the digital revolution. Br J Psychiatry.

[16] Lal S, Adair CE (2014). E-mental health: a rapid review of the literature. Psychiatr Serv.

[17] Riper H, Andersson G, Christensen H, Cuijpers P, Lange A, Eysenbach G (2010). Theme issue on e-mental health: a growing field in internet research. J Med Internet Res.

[18] Ebert DD, Zarski A, Christensen H, Stikkelbroek Y, Cuijpers P, Berking M, Riper H (2015). Internet and computer-based cognitive behavioral therapy for anxiety and depression in youth: a meta-analysis of randomized controlled outcome trials. PLoS One.

[19] Farrer L, Gulliver A, Chan JKY, Batterham PJ, Reynolds J, Calear A, Tait R, Bennett K, Griffiths KM (2013). Technology-based interventions for mental health in tertiary students: systematic review. J Med Internet Res.

[20] Huguet A, Rao S, McGrath PJ, Wozney L, Wheaton M, Conrod J, Rozario S (2016). A Systematic Review of Cognitive Behavioral Therapy and Behavioral Activation Apps for Depression. PLoS One.

[21] Pennant ME, Loucas CE, Whittington C, Creswell C, Fonagy P, Fuggle P, Kelvin R, Naqvi S, Stockton S, Kendall T, Expert AG (2015). Computerised therapies for anxiety and depression in children and young people: a systematic review and meta-analysis. Behav Res Ther.

[22] Reyes-Portillo JA, Mufson L, Greenhill LL, Gould MS, Fisher PW, Tarlow N, Rynn MA (2014). Web-based interventions for youth internalizing problems: a systematic review. J Am Acad Child Adolesc Psychiatry.

[23] Greenhalgh T, Robert G, Macfarlane F, Bate P, Kyriakidou O (2004). Diffusion of innovations in service organizations: systematic review and recommendations. Milbank Q.

[24] Kazdin AE, Blase SL (2011). Rebooting Psychotherapy Research and Practice to Reduce the Burden of Mental Illness. Perspect Psychol Sci.

[25] Li J, Talaei-Khoei A, Seale H, Ray P, Macintyre CR (2013). Health Care Provider Adoption of eHealth: Systematic Literature Review. Interact J Med Res.

[26] World Health Organization.

[27] Lyon AR, Wasse JK, Ludwig K, Zachry M, Bruns EJ, Unützer J, McCauley E (2016). The Contextualized Technology Adaptation Process (CTAP): Optimizing Health Information Technology to Improve Mental Health Systems. Adm Policy Ment Health.

[28] Mair FS, May C, O'Donnell C, Finch T, Sullivan F, Murray E (2012). Factors that promote or inhibit the implementation of e-health systems: an explanatory systematic review. Bull World Health Organ.

[29] Ross J, Stevenson F, Lau R, Murray E (2016). Factors that influence the implementation of e-health: a systematic review of systematic reviews (an update). Implement Sci.

[30] Moher D, Liberati A, Tetzlaff J, Altman DG (2010). Preferred reporting items for systematic reviews and meta-analyses: the PRISMA statement. Int J Surg.

[31] Proctor E, Silmere H, Raghavan R, Hovmand P, Aarons G, Bunger A, Griffey R, Hensley M (2011). Outcomes for implementation research: conceptual distinctions, measurement challenges, and research agenda. Adm Policy Ment Health.

[32] Altman D (1991). Practical statistics for medical research. Practical statistics for medical research.

[33] Pluye P, Robert E, Cargo M, Bartlett G, O'Cathain A, Griffiths F (2011). McGill University.

[34] Pace R, Pluye P, Bartlett G, Macaulay AC, Salsberg J, Jagosh J, Seller R (2012). Testing the reliability and efficiency of the pilot Mixed Methods Appraisal Tool (MMAT) for systematic mixed studies review. Int J Nurs Stud.

[35] Crabtree B, Miller W (1992). A template approach to text analysis: developing and using codebooks. Research methods for primary care.

[36] Morgan AJ, Rapee RM, Bayer JK (2016). Prevention and early intervention of anxiety problems in young children: A pilot evaluation of Cool Little Kids Online. Internet Interventions.

[37] Crawford EA, Salloum A, Lewin AB, Andel R, Murphy TK, Storch EA (2013). A Pilot Study of Computer-Assisted Cognitive Behavioral Therapy for Childhood Anxiety in Community Mental Health Centers. J Cogn Psychother.

[38] Khanna MS, Kendall PC (2010). Computer-assisted cognitive behavioral therapy for child anxiety: results of a randomized clinical trial. J Consult Clin Psychol.

[39] Salloum A, Crawford EA, Lewin AB, Storch EA (2015). Consumers' and providers' perceptions of utilizing a computer-assisted cognitive behavioral therapy for childhood anxiety. Behav Cogn Psychother.

[40] Salloum A, Johnco C, Lewin AB, McBride NM, Storch EA (2016). Barriers to access and participation in community mental health treatment for anxious children. J Affect Disord.

[41] Storch EA, Salloum A, King MA, Crawford EA, Andel R, McBride NM, Lewin AB (2015). A Randomized Controlled Trial In Community Mental Health Centers Of Computer-Assisted Cognitive Behavioral Therapy Versus Treatment As Usual For Children With Anxiety. Depress Anxiety.

[42] Vigerland S, Ljótsson B, Thulin U, Öst L, Andersson G, Serlachius E (2016). Internet-delivered cognitive behavioural therapy for children with anxiety disorders: A randomised controlled trial. Behav Res Ther.

[43] Reuland MM, Teachman BA (2014). Interpretation bias modification for youth and their parents: a novel treatment for early adolescent social anxiety. J Anxiety Disord.

[44] Cunningham M, Wuthrich V (2008). Examination of Barriers to Treatment and User Preferences With Computer-based Therapy Using The Cool Teens CD for Adolescent Anxiety. EJAP.

[45] Pramana G, Parmanto B, Kendall PC, Silk JS (2014). The SmartCAT: an m-health platform for ecological momentary intervention in child anxiety treatment. Telemed J E Health.

[46] Donovan CL, March S (2014). Online CBT for preschool anxiety disorders: a randomised control trial. Behav Res Ther.

[47] Anderson REE, Spence SH, Donovan CL, March S, Prosser S, Kenardy J (2012). Working alliance in online cognitive behavior therapy for anxiety disorders in youth: comparison with clinic delivery and its role in predicting outcome. J Med Internet Res.

[48] Spence SH, Donovan CL, March S, Gamble A, Anderson RE, Prosser S, Kenardy J (2011). A randomized controlled trial of online versus clinic-based CBT for adolescent anxiety. J Consult Clin Psychol.

[49] March S, Spence SH, Donovan CL (2009). The efficacy of an internet-based cognitive-behavioral therapy intervention for child anxiety disorders. J Pediatr Psychol.

[50] Spence SH, Holmes JM, March S, Lipp OV (2006). The feasibility and outcome of clinic plus internet delivery of cognitive-behavior therapy for childhood anxiety. J Consult Clin Psychol.

[51] Brezinka V (2013). Ricky and the spider - a video game to support cognitive behavioural treatment of children with obsessive-compulsive disorder. Clinical Neuropsychiatry.

[52] Cunningham M, Rapee R, Lyneham H (2006). Feedback to a prototype self-help computer program for anxiety disorders in adolescents. Aust E J Adv Ment Health.

[53] Wuthrich VM, Rapee RM, Cunningham MJ, Lyneham HJ, Hudson JL, Schniering CA (2012). A randomized controlled trial of the Cool Teens CD-ROM computerized program for adolescent anxiety. J Am Acad Child Adolesc Psychiatry.

[54] Tillfors M, Andersson G, Ekselius L, Furmark T, Lewenhaupt S, Karlsson A, Carlbring P (2011). A randomized trial of Internet-delivered treatment for social anxiety disorder in high school students. Cogn Behav Ther.

[55] Sarver NW, Beidel DC, Spitalnick JS (2014). The feasibility and acceptability of virtual environments in the treatment of childhood social anxiety disorder. J Clin Child Adolesc Psychol.

[56] Simmons MB, Elmes A, McKenzie JE, Trevena L, Hetrick SE (2017). Right choice, right time: Evaluation of an online decision aid for youth depression. Health Expect.

[57] Hetrick SE, Dellosa MK, Simmons MB, Phillips L (2015). Development and pilot testing of an online monitoring tool of depression symptoms and side effects for young people being treated for depression. Early Interv Psychiatry.

[58] Hetrick SE, Goodall J, Yuen HP, Davey CG, Parker AG, Robinson J, Rickwood DJ, McRoberts A, Sanci L, Gunn J, Rice S, Simmons MB (2017). Comprehensive Online Self-Monitoring to Support Clinicians Manage Risk of Suicide in Youth Depression. Crisis.

[59] Rice S, Gleeson J, Davey C, Hetrick S, Parker A, Lederman R, Wadley G, Murray G, Herrman H, Chambers R, Russon P, Miles C, D'Alfonso S, Thurley M, Chinnery G, Gilbertson T, Eleftheriadis D, Barlow E, Cagliarini D, Toh J, McAlpine S, Koval P, Bendall S, Jansen JE, Hamilton M, McGorry P, Alvarez-Jimenez M (2016). Moderated online social therapy for depression relapse prevention in young people: pilot study of a 'next generation' online intervention. Early Interv Psychiatry.

[60] Ranney ML, Freeman JR, Connell G, Spirito A, Boyer E, Walton M, Guthrie KM, Cunningham RM (2016). A Depression Prevention Intervention for Adolescents in the Emergency Department. J Adolesc Health.

[61] Kobak KA, Mundt JC, Kennard B (2015). Integrating technology into cognitive behavior therapy for adolescent depression: a pilot study. Ann Gen Psychiatry.

[62] Davidson TM, Yuen EK, Felton JW, McCauley J, Gros KS, Ruggiero KJ (2014). Feasibility assessment of a brief, web-based behavioral activation intervention for adolescents with depressed mood. Int J Psychiatry Med.

[63] Van Voorhees BW, Fogel J, Pomper BE, Marko M, Reid N, Watson N, Larson J, Bradford N, Fagan B, Zuckerman S, Wiedmann P, Domanico R (2009). Adolescent Dose and Ratings of an Internet-Based Depression Prevention Program: A Randomized Trial of Primary Care Physician Brief Advice versus a Motivational Interview. J Cogn Behav Psychother.

[64] Van Voorhees BW, Fogel J, Reinecke MA, Gladstone T, Stuart S, Gollan J, Bradford N, Domanico R, Fagan B, Ross R, Larson J, Watson N, Paunesku D, Melkonian S, Kuwabara S, Holper T, Shank N, Saner D, Butler A, Chandler A, Louie T, Weinstein C, Collins S, Baldwin M, Wassel A, Vanderplough-Booth K, Humensky J, Bell C (2009). Randomized clinical trial of an Internet-based depression prevention program for adolescents (Project CATCH-IT) in primary care: 12-week outcomes. J Dev Behav Pediatr.

[65] Van Voorhees BW, Vanderplough-Booth K, Fogel J, Gladstone T, Bell C, Stuart S, Gollan J, Bradford N, Domanico R, Fagan B, Ross R, Larson J, Watson N, Paunesku D, Melkonian S, Kuwabara S, Holper T, Shank N, Saner D, Butler A, Chandler A, Louie T, Weinstein C, Collins S, Baldwin M, Wassel A, Reinecke MA (2008). Integrative internet-based depression prevention for adolescents: a randomized clinical trial in primary care for vulnerability and protective factors. J Can Acad Child Adolesc Psychiatry.

[66] Van Voorhees BW, Watson N, Bridges JFP, Fogel J, Galas J, Kramer C, Connery M, McGill A, Marko M, Cardenas A, Landsback J, Dmochowska K, Kuwabara SA, Ellis J, Prochaska M, Bell C (2010). Development and pilot study of a marketing strategy for primary care/internet-based depression prevention intervention for adolescents (the CATCH-IT intervention). Prim Care Companion J Clin Psychiatry.

[67] Ruby A, Marko-Holguin M, Fogel J, Van Voorhees BW (2013). Economic analysis of an internet-based depression prevention intervention. J Ment Health Policy Econ.

[68] Iloabachie C, Wells C, Goodwin B, Baldwin M, Vanderplough-Booth K, Gladstone T, Murray M, Fogel J, Van Voorhees BW (2011). Adolescent and parent experiences with a primary care/Internet-based depression prevention intervention (CATCH-IT). Gen Hosp Psychiatry.

[69] Gladstone T, Marko-Holguin M, Henry J, Fogel J, Diehl A, Van Voorhees BW (2014). Understanding adolescent response to a technology-based depression prevention program. J Clin Child Adolesc Psychol.

[70] Eisen JC, Marko-Holguin M, Fogel J, Cardenas A, Bahn M, Bradford N, Fagan B, Wiedmann P, Van Voorhees BW (2013). Pilot Study of Implementation of an Internet-Based Depression Prevention Intervention (CATCH-IT) for Adolescents in 12 US Primary Care Practices: Clinical and Management/Organizational Behavioral Perspectives. Prim Care Companion CNS Disord.

[71] Merry SN, Stasiak K, Shepherd M, Frampton C, Fleming T, Lucassen MFG (2012). The effectiveness of SPARX, a computerised self help intervention for adolescents seeking help for depression: randomised controlled non-inferiority trial. BMJ.

[72] Demaso DR, Marcus NE, Kinnamon C, Gonzalez-Heydrich J (2006). Depression experience journal: a computer-based intervention for families facing childhood depression. J Am Acad Child Adolesc Psychiatry.

[73] Sapru I, Khalid-Khan S, Choi E, Alavi N, Patel A, Sutton C, Odejayi G, Calancie OG (2016). Effectiveness of online versus live multi-family psychoeducation group therapy for children and adolescents with mood or anxiety disorders: a pilot study. Int J Adolesc Med Health.

[74] Brezinka V (2014). Computer games supporting cognitive behaviour therapy in children. Clin Child Psychol Psychiatry.

[75] Bobier C, Stasiak K, Mountford H, Merry S, Moor S (2014). When ‘e’ therapy enters the hospital: Examination of the feasibility and acceptability of SPARX (a cCBT programme) in an adolescent inpatient unit. Advances in Mental Health.

[76] Hoek W, Schuurmans J, Koot HM, Cuijpers P (2012). Effects of Internet-based guided self-help problem-solving therapy for adolescents with depression and anxiety: a randomized controlled trial. PLoS One.

[77] Ercan S (2006). Evaluation of a mental health website for teenagers. Psychiatric Bulletin.

[78] Lenhard F, Vigerland S, Andersson E, Rück C, Mataix-Cols D, Thulin U, Ljótsson B, Serlachius E (2014). Internet-delivered cognitive behavior therapy for adolescents with obsessive-compulsive disorder: an open trial. PLoS One.

[79] Vigerland S, Thulin U, Ljótsson B, Svirsky L, Ost L, Lindefors N, Andersson G, Serlachius E (2013). Internet-delivered CBT for children with specific phobia: a pilot study. Cogn Behav Ther.

[80] Lenhard F, Vigerland S, Engberg H, Hallberg A, Thermaenius H, Serlachius E (2016). “On My Own, but Not Alone” - Adolescents' Experiences of Internet-Delivered Cognitive Behavior Therapy for Obsessive-Compulsive Disorder. PLoS One.

[81] Carrasco A (2016). Acceptability of an adventure video game in the treatment of female adolescents with symptoms of depression. Research in Psychotherapy: Psychopathology, Process and Outcome.

[82] Struthers A, Charette C, Bapuji SB, Winters S, Ye X, Metge C, Kreindler S, Raynard M, Lemaire J, Synyshyn M, Sutherland K (2015). The Acceptability of E-mental Health Services for Children, Adolescents, and Young Adults: A Systematic Search and Review. Canadian Journal of Community Mental Health.

[83] Madan A, Sharp C, Newlin E, Vanwoerden S, Fowler JC (2016). Adolescents Are Less Satisfied with Inpatient Psychiatric Care than Their Parents: Does It Matter?. J Healthc Qual.

[84] Christensen H, Griffiths K, Farrer L (2011). Adherence in internet interventions for anxiety and depression. Journal of Medical Internet Research.

[85] Cheung KL, Ten KPM, Smit C, de VH, Pieterse ME (2017). The impact of non-response bias due to sampling in public health studies: A comparison of voluntary versus mandatory recruitment in a Dutch national survey on adolescent health. BMC Public Health.

[86] Kalyanaraman S, Sundar SS (2006). The Psychological Appeal of Personalized Content in Web Portals: Does Customization Affect Attitudes and Behavior?. J Communication.

[87] John J (1992). Patient satisfaction: the impact of past experience. J Health Care Mark.

[88] Michie S, Johnston M, Abraham C, Lawton R, Parker D, Walker A (2005). Making psychological theory useful for implementing evidence based practice: a consensus approach. Qual Saf Health Care.

[89] Eccles MP, Hrisos S, Francis J, Kaner EF, Dickinson HO, Beyer F, Johnston M (2006). Do self- reported intentions predict clinicians' behaviour: a systematic review. Implement Sci.

[90] Kuo Y, Walker AE, Belland BR, Schroder KEE (2013). A predictive study of student satisfaction in online education programs. Int Rev of Res in Open and Dis Learn.

[91] Montague AE, Varcin KJ, Simmons MB, Parker AG (2015). Putting Technology Into Youth Mental Health Practice: Young People's Perspectives. SAGE Open.

[92] Scheid TL (1994). An explication of treatment ideology among mental health care providers. Sociol Health & Illness.

[93] Baker-Ericzén MJ, Jenkins MM, Haine-Schlagel R (2013). Therapist, Parent, and Youth Perspectives of Treatment Barriers to Family-Focused Community Outpatient Mental Health Services. J Child Fam Stud.

[94] Flewett T (2010). Clinical Risk Management: An introductory text for mental health clinicians.

[95] Laxman K, Krishnan S, Dhillon J (2015). Barriers to adoption of consumer health informatics applications for health self management. Health Science Journal.

[96] Sarvet B, Torous J (2016). Health information technology for child and adolescent psychiatry. Child and Adolescent Psychiatric Clinics of North America.

[97] Broens THF, Huis IVRMHA, Vollenbroek-Hutten MMR, Hermens HJ, van HAT, Nieuwenhuis LJM (2007). Determinants of successful telemedicine implementations: a literature study. J Telemed Telecare.

[98] Lewis CC, Fischer S, Weiner BJ, Stanick C, Kim M, Martinez RG (2015). Outcomes for implementation science: an enhanced systematic review of instruments using evidence-based rating criteria. Implement Sci.

[99] Reynolds J, Griffiths KM, Cunningham JA, Bennett K, Bennett A (2015). Clinical Practice Models for the Use of E-Mental Health Resources in Primary Health Care by Health Professionals and Peer Workers: A Conceptual Framework. JMIR Ment Health.

[100] Damschroder LJ, Aron DC, Keith RE, Kirsh SR, Alexander JA, Lowery JC (2009). Fostering implementation of health services research findings into practice: a consolidated framework for advancing implementation science. Implement Sci.

[101] Durlak JA, DuPre EP (2008). Implementation matters: a review of research on the influence of implementation on program outcomes and the factors affecting implementation. Am J Community Psychol.

[102] Proctor EK, Landsverk J, Aarons G, Chambers D, Glisson C, Mittman B (2009). Implementation research in mental health services: an emerging science with conceptual, methodological, and training challenges. Adm Policy Ment Health.

[103] Curran GM, Bauer M, Mittman B, Pyne JM, Stetler C (2012). Effectiveness-implementation hybrid designs: combining elements of clinical effectiveness and implementation research to enhance public health impact. Med Care.

[104] Cohen DJ, Crabtree BF, Etz RS, Balasubramanian BA, Donahue KE, Leviton LC, Clark EC, Isaacson NF, Stange KC, Green LW (2008). Fidelity versus flexibility: translating evidence-based research into practice. Am J Prev Med.

[105] Dixon B, Zafar A, McGowan J (2007). Development of a taxonomy for health information technology. Studies in Health Technology and Informatics.

[106] Oh H, Rizo C, Enkin M, Jadad A (2005). What is eHealth?: a systematic review of published definitions. World Hosp Health Serv.

[107] Pagliari C, Sloan D, Gregor P, Sullivan F, Detmer D, Kahan JP, Oortwijn W, MacGillivray S (2005). What is eHealth (4): a scoping exercise to map the field. J Med Internet Res.

