# Supplementary material for: eMental Healthcare Technologies for Anxiety and Depression in Childhood and Adolescence: Systematic Review of Studies Reporting Implementation Outcomes
Source: JMIR Ment Health. 2018 Jun 26;5(2):e48. doi: 10.2196/mental.9655 (PMC6039769; doi:10.2196/mental.9655)
Supplement: Multimedia Appendix 1 [file mental_v5i2e48_app1.pdf]

1. ((electronic or mobile) adj3 (mental health or MH or psychiatr*)).tw,kf. (199)
2. (e-mental health* or emental health*).tw,kf. (119)
3. (e-psychiatr* or epsychiatr*).tw,kf. (9)
4. (m-mental health* or mmental health*).tw,kf. (7)
5. (tele-psychiatr* or telepsychiatr*).tw,kf. (474)
6. (tele-mental health* or telemental health*).tw,kf. (137)
7. or/1-6 [Combined coordinated concepts for e-mental health] (893)
8. exp Cell Phones/ (8925)
9. Computers/ (51348)
10. exp Computers, Handheld/ (3887)
11. Electronic Mail/ (2539)
12. exp Internet/ (70085)
13. Microcomputers/ (14765)
14. Mobile Applications/ (1600)
15. Remote Consultation/ (4285)
16. exp Telemedicine/ (20996)
17. Therapy, Computer-Assisted/ (6392)
18. Video Recording/ (21952)
19. Videoconferencing/ (1146)
20. Virtual Reality Exposure Therapy/ (328)
21. (android* or apps or cell* phone or cellphone* or cell* tele-phone* or cell* telephone* or iPad* or iPhone* or iPod* or smart phone* or smartphone*).tw,kf. (11189)
22. blog*.tw,kf. (1278)
23. (chat or chatroom*).tw,kf. (6112)
24. (communicat* adj2 technolog*).tw,kf. (3677)
25. computer*.ti,kf. (75443)
26. computer*.ab./freq=2 (43951)
27. digital*.ti,kf. (38707)
28. digital*.ab./freq=2 (22967)
29. (e-Health* or eHealth*).tw,kf. (3583)
30. (e-mail* or electronic mail* or email*).tw,kf. (12717)
31. (e-medic* or emedic*).tw,kf. (188)
32. (e-therap* or etherap*).tw,kf. (699)
33. electronic communic*.tw,kf. (1168)
34. electronic health*.tw,kf. (10577)
35. facebook*.tw,kf. (1937)
36. (information adj2 technolog*).tw,kf. (12686)
37. internet*.ti,kf. (15209)
38. internet*.ab./freq=2 (11976)
39. (m-health* or mhealth* or mobile health*).tw,kf. (2876)
40. media technolog*.tw,kf. (170)
41. (mobile adj2 (app* or device* or phone* or technolog* or tele-phone* or telephone*)).tw,kf. (10475)
42. (on-line* or online*).ti,kf. (22885)
43. (on-line* or online*).ab./freq=2 (19250)
44. (PDA* or personal digital assistant*).tw,kf. (13481)

45. (portable adj2 (app* or device* or phone* or tele-phone* or telephone*)).tw,kf. (2809)
46. (short messag* or sms).tw,kf. (4740)
47. social media.tw,kf. (4437)
48. (tele-care* or telecare*).tw,kf. (637)
49. (tele-health* or telehealth*).tw,kf. (3033)
50. (tele-medic* or telemedic*).tw,kf. (9032)
51. (tele-p?ediatric* or telep?ediatric*).tw,kf. (26)
52. text messag*.tw,kf. (2505)
53. (tweet* or twitter).tw,kf. (1960)
54. virtual*.ti,kf. (17229)
55. virtual*.ab./freq=2 (14652)
56. virtual reality.tw,kf. (6583)
57. web*.ti,kf. (25926)
58. web*.ab./freq=2 (25339)
59. wireless*.tw,kf. (9669)
60. or/8-59 [Combined MeSH & textwords for telemedicine and electronic applications] (445366)
61. exp Anxiety/ (72694)
62. Depression/ (102503)
63. exp Depressive Disorder/ (102650)
64. *Mental Disorders/th [Therapy] (21455)
65. *Mental Health Services/ (23596)
66. (affective adj2 (disorder* or dis regulat* or disregulat* or dys regulat* or dysregulation)).tw,kf. (16762)
67. (((anankastic or obsess* compulsiv*) adj1 (behavio* or disorder* or neuros* or personalit*)) or OCD).tw,kf. (14725)
68. anxi*.tw,kf. (172670)
69. (((behavio* or disorder* or episod*) adj1 (hypomanic or manic)) or mania*).tw,kf. (11434)
70. (bi-polar* or bipolar*).tw,kf. (58714)
71. cyclothymic disorder*.tw,kf. (95)
72. depress*.tw,kf. (410219)
73. (disruptive mood adj1 (dis regulat* or disregulat* or dys regulat* or dysregulat*)).tw,kf. (86)
74. dysthymi*.tw,kf. (3178)
75. mood disorder*.tw,kf. (15387)
76. (mood adj2 lability).tw,kf. (218)
77. (panic* adj1 (attack* or disorder*)).tw,kf. (11019)
78. (phobia* or phobic).tw,kf. (11035)
79. stress disorder*.tw,kf. (24847)
80. unstable mood*.tw,kf. (38)
81. or/61-80 [Combined MeSH & textwords for depression/anxiety] (679614)
82. and/60,81 [Combined concepts for telemedicine and electronic applications & depression/anxiety] (11858)
83. or/7,82 [Combined search sets for e-mental health & telemedicine and electronic applications & depression/anxiety] (12402)
84. Adolescent/ (1893735)
85. Adolescent Health/ (249)

86. Adolescent Health Services/ (5357)
87. Adolescent Medicine/ (1518)
88. Child/ (1593659)
89. Minors/ (2527)
90. exp Pediatrics/ (56693)
91. (adolescen* or boy* or girl* or minors or teen* or youth or youths).tw,jw,kf. (500045)
92. (child* or kid or kids or school age* or schoolage* or schoolchild*).tw,jw,kf. (1363046)
93. (elementary school* or high school* or highschool* or kindergar* or primary school* or secondary school*).tw,jw,kf. (58003)
94. p?ediatric*.tw,jw,kf. (651690)
95. or/84-94 [Combined MeSH & textwords for adolescents & children] (3451755)
96. and/83,95 [Combined concepts for e-mental health & adolescents/children] (3190)
97. exp Animals/ not Humans/ (4669479)
98. (animal or animal-model* or animals or canine* or cat or cats or dog or dogs or feline or felines or hamster or hamsters or mice or monkey or monkeys or mouse or pig or piglet or piglets or pigs or porcine or primate* or rabbit or rabbits or rat or rats or rodent or rodents or sheep or swine or swines).ti. (2061744)
99. 96 not (97 or 98) [Animal studies excluded] (3172)
100. case reports.pt. (1878769)
101. (case report* or case stud*).ti. (241676)
102. 99 not (100 or 101) [Case reports excluded] (3087)
103. (comment or editorial or news or newspaper article).pt. (1226424)
104. (comment* or editor* or news*).ti. (135992)
105. 102 not (103 or 104) [Opinion pieces excluded] (3045)
106. limit 105 to yr="2000-Current" (2852)
107. remove duplicates from 106 (2433)
