# Supplementary material for: eMental Healthcare Technologies for Anxiety and Depression in Childhood and Adolescence: Systematic Review of Studies Reporting Implementation Outcomes
Source: JMIR Ment Health. 2018 Jun 26;5(2):e48. doi: 10.2196/mental.9655 (PMC6039769; doi:10.2196/mental.9655)
Supplement: Multimedia Appendix 2 [file mental_v5i2e48_app2.pdf]

Multimedia Appendix. Study Quality according to Mixed Methods Appraisal Tool (MMAT).

**Qualitative studies**					
**Author**	**1.1**	**1.2**	**1.3**	**1.4**	**MMAT score**
	Are the sources of qualitative data relevant to address the question?	Is the process for analyzing data relevant to address the question?	Is consideration given to how findings relate to the context?	Is consideration given to how findings relate to researcher influence?	
Lenhard et al. [80]	□	□	□	□	100
**Quantitative randomized controlled trials**					
**Author**	**2.1**	**2.2**	**2.3**	**2.4**	**MMAT score**
	Is there a clear description of the randomization or appropriate sequence generation?	Is there a clear description of the allocation concealment?	Are there complete outcome data (80% or above)?	Is there low withdrawal/drop-out (below 20%)?	
Donovan et al. [46]	□	□	□	□	100
Anderson et al. [47]	□	□	□	□	100
Merry et al. [71]	□	□	□	□	100
Spence et al. [48]	□	□	□	□	100
March et al. [49]	□	□	□	□	100
Van Voorhees et al. [63]	□	□	□	□	100
Van Voorhees et al. [64]	□	□	□	□	100
Van Voorhees et al. [65]	□	□	□	□	100
Spence et al. [50]	□	□	□	□	100
Morgan et al. [36]	□	x	□	□	75
Wuthrich et al. [53]	□	x	□	□	75
Khanna et al.	□	x	□	□	75

[38]					
Reuland et al. [43]	x	x	✓	✓	50
Tillfors et al. [54]	x	x	✓	✓	50
Vigerland et al. [42]	x	x	✓	✓	50
Kobak et al. [61]	x	x	✓	✓	50
Gladstone et al. [69]	x	x	✓	✓	50
Hoek et al. [76]	✓	✓	x	x	50
Storch et al. [41]	✓	x	✓	x	25
Salloum et al. [40]	x	✓	x	x	25
Eisen et al. [70]	x	x	o	o	0
**Quantitative non-randomized trials**					
**Author**	**3.1**	**3.2**	**3.3**	**3.4**	**MMAT score**
	Are participants recruited in a way that minimizes selection bias?	Are measurements appropriate regarding the exposure / intervention, outcomes?	In the groups being compared, are the participants comparable, or do researchers take into account the difference between these groups?	Are there complete outcome data (80% or above), and, when applicable, an acceptable response rate (60% or above), or an acceptable follow-up rate for cohort studies (depending on the duration of follow-up)?	
Sapru et al. [73]	x	✓	✓	x	50
**Quantitative descriptive studies**					
**Author**	**4.1**	**4.2**	**4.3**	**4.4**	**MMAT score**
	Is the sampling strategy relevant to address the research question?	Is the sample representative of the population under study?	Are measurements appropriate?	Is there an acceptable response rate (60% or above)?	
Carrasco et al. [81]	✓	✓	✓	✓	100
Rice et al. [59]	✓	✓	✓	✓	100
Simmons et al. [56]	✓	✓	✓	✓	100
Lenhard et al.	✓	✓	✓	✓	100

[78]					
Sarver et al. [55]	✓	✓	✓	✓	100
Bobier et al. [75]	✓	✓	✓	✓	100
Crawford et al. [37]	✓	✓	✓	✓	100
Ruby et al. [67]	✓	✓	✓	✓	100
Vigerland et al. [79]	✓	✓	✓	✓	100
Van Voorhees et al. [66]	✓	✓	✓	✓	100
Ercan et al. [77]	✓	✓	✓	✓	100
Cunningham et al. [52]	✓	✓	✓	✓	100
Brezinka et al. [51]	✓	✓	o	✓	75
Brezinka et al. [74]	✓	✓	o	✓	75
Pramana et al. [45]	o	✓	✓	o	50
Hetrick et al. [58]	✓	✓	x	x	50

**Mixed methods studies**																
**Author**	**1.1**	**1.2**	**1.3**	**1.4**	**3.1**	**3.2**	**3.3**	**3.4**	**4.1**	**4.2**	**4.3**	**4.4**	**5.1**	**5.2**	**5.3**	**MMAT score**
	Are the sources of qualitative data relevant to address the question?	Is the process for analyzing data relevant to address the question?	Is consideration given to how findings relate to the context?	Is consideration given to how findings relate to researcher influence?	Are participants recruited in a way that minimizes selection bias?	Are measurements appropriate regarding the exposure / intervention, outcomes?	In the groups being compared, are the participants comparable, or do researchers take into account the difference between these groups?	Are there complete outcome data (80% or above), and, when applicable, an acceptable response rate (60% or above), or an acceptable follow-up rate for cohort studies (depending on	Is the sampling strategy relevant to address the research question?	Is the sample representative of the population under study?	Are measurements appropriate?	Is there an acceptable response rate (60% or above)?	Is the design relevant to address the question?	Is the integration of data / results relevant to address the question?	Is consideration given to the limitations associated with this integration in a triangulation design?	

								the duration of follow-up)?								
Hetrick et al. [57]	✓	✓	x	✓					✓	✓	✓	✓	✓	✓	✓	100
Salloum et al. [39]	✓	✓	✓	x					✓	✓	✓	✓	✓	✓	x	75
Cunningham et al. [44]	✓	✓	✓	✓					✓	✓	✓	✓	✓	✓	x	75
Ranney et at. [60]	✓	✓	✓	x					✓	✓	✓	✓	✓	✓	✓	75
Iloabachie et al. [68]	✓	✓	✓	✓					✓	✓	✓	x	✓	✓	✓	75
Demaso et al. [72]	✓	✓	x	x					✓	✓	✓	x	✓	✓	x	50
Davidson et al. [62]	✓	✓	x	x					x	x	x	✓	✓	✓	x	25

✓=yes; x=no; o=can't tell; RCT: Randomized Controlled Trial; MMAT=Mixed Methods Appraisal Tool; All studies undergoing quality assessment passed the screening questions: 1) Clear research question? 2) Do collected data address the question?
